# LncRNA FAM66C enhances gastric cancer proliferation and chemoresistance through phosphorylating nuclear YAP protein 

**DOI:** 10.3389/fphar.2026.1749191

**Published:** 2026-07-30

**Authors:** Chao Yang, Hai-Lan Guan, Li-Hua Ji, Gang-Qiang Wang, Li-Si Zeng, Xian-Zi Yang

**Affiliations:** 1 Department of Medical Oncology, Guangzhou Institute of Cancer Research, the Affiliated Cancer Hospital, Guangzhou Medical University, Guangzhou, China; 2 Department of Electrocardiogram, Guangzhou Institute of Cancer Research, the Affiliated Cancer Hospital, Guangzhou Medical University, Guangzhou, China; 3 Guangzhou Institute of Cancer Research, the Affiliated Cancer Hospital, Guangzhou Medical University, Guangzhou, China

**Keywords:** chemoresistance, FAM66C, gastric cancer, prognosis, YAP phosphorylation

## Abstract

**Background:**

Chemoresistance is one of the causes of death in patients with gastric cancer (GC). Extensive studies have demonstrated that lncRNAs have important implications in GC chemoresistance, but their underlying mechanisms in driving GC chemoresistance remain poorly understood. Here, we investigated the function and molecular mechanism of lncRNA FAM66C in GC progression and chemoresistance.

**Methods:**

After long-term exposure to cisplatin (DDP) or paclitaxel (PTX), we successfully established two chemoresistance GC cell lines, HGC-27/DDP and HGC-27/PTX. The expression and biological function of FAM66C in GC cells were assessed using RT-PCR, drug sensitivity (IC50), cell viability (CCK8), apoptosis assay and *in vivo* xenograft models. The underlying mechanism of this lncRNA in GC chemoresistance was determined by Western blotting, RNA-FISH assay, RNA immunoprecipitation assay and rescue experiments.

**Results:**

FAM66C overexpression was observed in GC tissues and its elevated expression positively correlated with chemotherapy resistance and poor patient prognosis. Functional studies confirmed that it promoted GC proliferation and chemoresistance *in vitro* and *in vivo*. FAM66C induced nuclear YAP phosphorylation by interacting with nuclear YAP in a Hippo pathway-independent manner.

**Conclusion:**

In this study, we demonstrated that FAM66C facilitated GC chemoresistance by promoting the phosphorylation of nuclear YAP independently of the Hippo pathway. This lncRNA might serve as a novel prognostic biomarker and a potential therapeutic target for overcoming GC chemoresistance.

## Introduction

As a common infection-related cancer, gastric cancer (GC) remains a leading health threat (Bray et al., 2024). There were 358,700 new cases of GC and 260,400 deaths in China in 2022, accounting for over one-third of total new GC cases and deaths worldwide (Han et al., 2024). Despite advances in immunotherapy and targeted therapy, systemic chemotherapy is still the cornerstone of treatment for advanced GC (AGC) (Sundar et al., 2025). However, GC cells repeatedly exposed to chemotherapy drugs eventually develop treatment resistance, leading to recurrence or metastasis (Zhan et al., 2025). Therefore, understanding the molecular mechanisms of chemoresistance and identifying new molecular targets are crucial for improving the therapeutic effect and reversing chemotherapy resistance.

During the last decades, significant progress has been made in elucidating the biological functions of long non-coding RNAs (lncRNAs), including epigenetics, gene transcription, RNA sponging and degradation (Singh et al., 2022). Accruing evidence points to their pivotal role in cancer progression, including cancer proliferation, apoptosis and treatment resistance (Hashemi et al., 2022). Many lncRNAs have been recently shown to exert critical roles in chemoresistance via regulating drug efflux, cell death machinery, DNA damage repair, autophagy and oxidative stress management, oncogenes and miRNAs, and so on (Eptaminitaki et al., 2022). Substantial evidence demonstrates that the dysregulated expression of lncRNAs is central to GC progression and drug resistance (Wu et al., 2022). For example, LINC01133 inhibited GC proliferation and migration by acting as a ceRNA for miR-106a-3p to regulate APC expression and Wnt/β-catenin pathway (Yang et al., 2018). Luo et al. (2021) found that lncRNA *EIF3J-DT* activated autophagy and contributed to GC cells chemoresistance by sponging miR188-3p via increasing ATG14 expression. Li et al. (2020) revealed that overexpression of lncRNA PCAT-1 induced cisplatin resistance in GC cells by epigenetically inhibiting PTEN through recruiting EZH2. These findings suggest that lncRNAs confer drug resistance in GC.

In our previous study, we successfully constructed a ceRNA network related to cisplatin chemoresistance in GC using RNA sequencing and bioinformatic analyses, and identified two potential chemoresistant-related lncRNAs (FAM66C and SFTA1P) in GC (Yang et al., 2022). Previous studies showed that FAM66C overexpression was found in lung cancer and intrahepatic cholangiocarcinoma (ICC) (Lei et al., 2021; Wang et al., 2023). FAM66C promoted ICC tumor progression and glycolytic activity by regulating miR-23b-3p/KCND2 axis (Lei et al., 2021). In prostate cancer, FAM66C enhanced cell proliferation by negative modulation of proteasome pathway to activate EGFR-ERK signaling (Xie et al., 2020). However, FAM66C expression was downregulated in glioma cells and inhibited glioma growth through miR15a(b)/LATS1 pathway (Xiao and Peng, 2022). The biological functions of FAM66C vary across different cancer types, suggesting that there is tissue-specific regulation of this lncRNA. However, the function and chemoresistant mechanism of FAM66C in GC have remained poorly understood.

In this study, we aim to investigate the expression and clinical significance of FAM66C in GC cells and tissues, along with its biological function in parental and chemoresistant GC cells. Moreover, we will explore the underlying mechanisms through which FAM66C regulates drug resistance.

## Materials and methods

### GC patients and tissue samples

This study consisted of 147 GC tumor and 28 surrounding normal tissues, which were obtained from at Guangzhou Institute of Cancer Research, the Affiliated Cancer Hospital of Guangzhou Medical University between 2007 and 2011. All GC patients had not received any local or systemic treatment before surgery. All tissue samples were snap-frozen immediately after surgery and stored at −80 °C for RNA extraction. The study was reviewed and approved by the medical ethics committee of Guangzhou Institute of Cancer Research, the Affiliated Cancer Hospital of Guangzhou Medical University, and informed consent was obtained from all patients.

### Cell culture and treatment

Human GC cell lines (HGC-27, AGS, MKN45, N87, SUN-1) and gastric mucosal epithelial cell line GES-1 were purchased from the Cell Bank of the Chinese Academy of Sciences (Shanghai, China). These cell lines were cultured in PRMI-1640 medium (Gibco, USA) supplemented with 10% fetal bovine serum (FBS, Procell, China) in a humidified incubator (37 °C, 5% CO2).

Cisplatin-resistant HGC-27 cells (HGC-27/DDP) and paclitaxel-resistant HGC-27 cells (HGC-27/PTX) were established by our laboratory. Briefly, HGC-27 cells were initially exposed to 0.5 μM of DDP or 0.005 μM of PTX for 2 weeks. The drug concentration was intermittently increased up to 15 μM of DDP or 1.5 μM of PTX over 1 year. To maintain the chemoresistant phenotype, these 2 cell lines were cultured in complete culture medium with 4 μM of DDP or 0.5 μM of PTX, respectively.

### RNA extraction and real-time quantitative PCR (RT-PCR)

The total RNA of GC tissues and cells was separated using TRIzol reagent (Invitrogen, Carlsbad, CA, USA). High-quality cDNA was synthesized by Prime-Script RT Reagent Kit (TaKaRa, Tokyo, Japan). RT-PCR was conducted using SYBR® Premix Ex Taq™ Kit (TaKaRa). The expression of lncRNAs and mRNAs were measured by the 2^−ΔΔCt^ method and normalized to the expression of GAPDH. Each sample was run in triplicate. The primer sequences used in this study are listed below: GAPDH, Forward 5′-CTC​CTC​CTG​TTT​CGA​CAG​TCA​GC-3′, Reverse 5′-CCC​AAT​ACG​ACC​AAA​TCC​GTT-3′; U6, Forward 5′-CTC​GCT​TCG​GCA​GCA​CA-3′, Reverse 5′-AAC​GCT​TCA​CGA​ATT​TGC​GT-3′; FAM66C, Forward 5′-CAT​CAC​TCA​CTC​TTC​CTG​GTC​T-3′, Reverse 5′-AGT​GGT​TCT​GTG​GCC​TCT​G-3′; YAP, Forward 5′-TAG​CCC​TGC​GTA​GCC​AGT​TA-3′, Reverse 5′-TCA​TGC​TTA​GTC​CAC​TGT​CTG​T-3′.

### Plasmid construction and cell transfection

The lentivirus-containing short hairpin RNAs (shRNAs) targeting FAM66C and YAP were designed and synthesized by GeneChem (Shanghai, China). FAM66C overexpression vector was purchased from iGeneBio (Guangzhou, China). According to the manufacturer’s instructions, GC cells were transfected using Lipofectamine 3000 (Invitrogen, Carlsbad, CA, USA), and cultured for another 48h and harvested for further experiments.

### CCK8 assay and IC50 cytotoxicity assay

Human GC cells were plated in 96-well plates at a density of 1.5–2.0 × 10^3^/well, and successively cultured for 7 days. Each well was added with medium with 10% CCK-8 reagents (GlpBio, USA) every day. The absorbance value of each sample was measured at a wavelength of 450 nm. For IC50 cytotoxicity assay, GC cells were seeded in 96-well plates and treated with a concentration gradient of cisplatin (DDP) or paclitaxel (PTX). Then 10 μL of CCK8 solution was added. Cell viability was observed by CCK8 assay. The IC50 values of each treatment group was defined as the dose at which DDP/PTX induces 50% cell death using Graphpad Prism 8.0.

### Colony formation assay and apoptosis assay

For colony formation assay, cells were seeded in 6-well plates (500 cells per well). After 14 days, the colonies were fixed with methanol and stained with 0.1% crystal violet, and then photographed. Apoptosis assay were performed using Annexin V-APC/7-AAD Apoptosis Kit (Multisciences, Hangzhou, China). The treated cells were re-suspended in 100 μL binding solution and stained with Annexin V-APC and 7-AAD at room temperature according to the manufacturer’s instructions. Cell apoptosis rates were measured by a BD FACSCanto II flow cytometry (BD Biosciences, USA).

### *In vivo* tumor growth assay and DDP-resistant mouse model

The protocol for animal experiments were approved by the Experimental Animal Ethics Committee of Shenzhen Lingfu Topu Biotechnology Co., Ltd. Female NCG mice, aged 4–6 weeks, were obtained from Guangdong GemPharmatech and maintained under specific pathogen-free conditions. For tumor xenograft model, HGC-27/DDP cells with or without FAM66C knockdown were separately injected into the right dorsal flank of NCG mice (5 × 10^6^ cells per 100 μL PBS). The length and width of each tumor was measured with calipers every 4 days, and the formula for calculating tumor volume was: (length × width^2^)/2. All mice were euthanized by cervical dislocation. The xenograft tumors were collected for immunohistochemistry (IHC) assay.

DDP-resistant mouse model was established to access the impact of FAM66C overexpression on GC chemoresistance *in vivo*. Briefly, HGC-27 cells (5 × 10^6^ cells per 100 μL PBS) stably transfected with lentivirus containing FAM66C sequence or empty vector were injected into the separately injected into the right dorsal flank of NCG mice. When the tumor volume reached 100 mm^3^, DDP (5 μg/mg) or normal saline (NS) was administered intraperitoneally every 3 days, for a total of 6 injections. One week after last administration, all mice were sacrificed and the tumors were collected for further analysis.

### Immunohistochemistry (IHC) analysis

Immunohistochemistry (IHC) experiment was conducted as previously described (Yang et al., 2018). Briefly, tumor tissues from mice were paraffin-embedded and cut into 4 μm thickness. These sections were deparaffinized, rehydrated and antigen retrieval. Then the sections were incubated with anti-Ki67 antibody (Proteintech, 1:4000 dilution, 27309-1-AP) at 4 °C overnight, followed by incubation with HRP-conjugated secondary antibody and then staining with DAB (Dako, Denmark). The immunostaining images were acquired and analyzed using a light microscope (Pannoramic 250, Tangier, China).

### Western blot analysis

The total or nuclear protein from GC cells were extracted using RIPA buffer (Beyotime, Shanghai, China). The quantified protein was separated by SDS-PAGE gels and transferred to PVDF membranes (Millipore, USA). The membranes were incubated with primary antibodies overnight at 4 °C. After incubation with an enzyme-linked secondary antibody, ECL detection reagents (Millipore) were used to observe the expression of proteins. The primary antibodies used in this assay included GAPDH, AKT, Phospho-AKT (Ser473), LATS1, Phospho-LATS1 (Thr1079), MOB1, Phospho-MOB1 (Thr35), mTOR, Phospho-mTOR (Ser2448), YAP, Phospho-YAP (Ser127), Phospho-YAP (Ser397) (Cell Signaling Technology), and Histone H3 (Proteintech). Protein band intensities were quantified by densitometry by ImageJ software and GAPDH served as a loading control.

### Subcellular fraction assay

The separation of cytosolic and nuclear fractions was conducted based on the protocol of the PARIS™ Kit (Thermo Fisher Scientific, USA). As per the product manuals, GC cells were collected and resuspended in cell fractionation buffer. Then the sample was centrifuged at 500 *g* for 5 min at 4 °C to isolate distinct subcellular fractions. RNA and protein extraction from the two fractions were subjected to following RT-PCR and Western blot assay, respectively.

### RNA immunoprecipitation (RIP) assay

RIP experiment was performed using EZ-Magna RIP™ Kit (Millipore, Bedford, USA) as instructed. Briefly, HGC-27 cells were lysed in RIPA lysis buffer and then incubated with RIP buffer, magnetic beads, and antibodies against YAP or negative control IgG antibody overnight at 4 °C. The co-precipitated RNAs were isolated, purified, and detected by RT-PCR analysis to demonstrate the fold enrichment of FAM66C. For the positive control, the anti-SNRNP70 antibody which pulls down the U1 snRNA, was applied.

### RNA fluorescence *in situ* hybridization (FISH) assay and immunofluorescence (IF) assay

LncRNA FAM66C-specific probes were designed and obtained from RiboBio (Guangzhou, China). GC cells were fixed in 4% paraformaldehyde (PFA) and then permeabilized with 0.5% Triton X-100. Cells were incubated with FAM66C-specific probes and primary antibodies against YAP or S127 YAP (Cell Signaling Technology). The nuclei were stained with 4′,6-diamidino-2-phenylindole dihydrochloride (DAPI; Life Technologies, Carlsbad CA, USA). All FISH and IF experiments were conducted according to the instructions of FISH kit (RiboBio, Guangzhou, China) and IF kit (RiboBio, Guangzhou, China). Images were captured by a confocal laser scanning microscope (Leica Microsystems, Germany).

### Statistical analysis

Statistical analyses were performed using SPSS 24.0 software (SPSS, Chicago, IL, USA) or GraphPad Prism V10 (GraphPad Prism, Inc., La Jolla, CA, USA). Kaplan-Meier analysis and the log-rank test were used to generate the survival curve and compare differences between survival curves, respectively. When variables had a normal distribution, Student’s t-test or one-way ANOVA was used for the comparison of two groups or more than two groups, respectively. If variables did not have a normal distribution, Mann-Whitney U test or Kruskal-Wallis test was used to compare the means of two or multiple groups. Two-tailed *P* ≤ 0.05 were considered to indicate statistical significance.

## Results

### FAM66C expression is elevated in GC tissues and associates with poor prognosis

**FIGURE 1:**
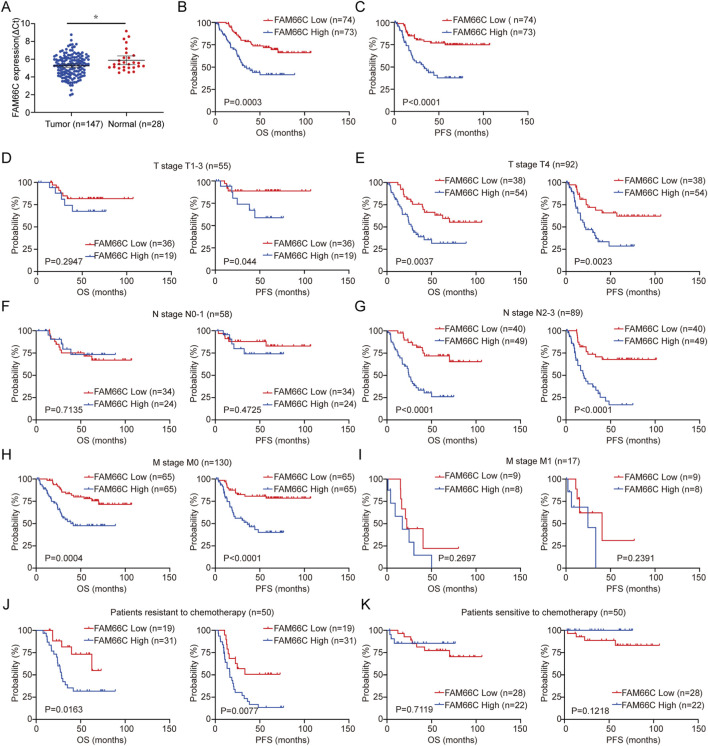
FAM66C expression was upregulated in GC tissues and positively associated with poor prognosis. **(A)** RT-PCR analyses of FAM66C expression in tumor tissues (n = 147) and surrounding normal tissues (n = 28) in GC patients. *P < 0.05. Results are presented as Δ cycle threshold (ΔCt) in tumor tissues relative to normal tissues. **(B,C)** Kaplan-Meier plots of the OS and PFS of all GC patients with high (n = 73) and low (n = 74) expression of FAM66C. The survival curve (OS, left panel; PFS, right panel) based on different FAM66C expression in GC patients with stage T1-3 and T4 **(D,E)**, patients with stage N0-1 and N2 **(F,G)**, patients with stage M0 and M1 **(H,I)**, patients resistant to or sensitive to chemotherapy **(J,K)**. Data were presented as mean ± SEM. P values were obtained by log-rank tests.

We first investigated the expression and prognostic significance of lncRNA FAM66C in GC tissues. The result showed that FAM66C expression was significantly higher in GC tissues than surrounding normal tissues (*P* < 0.05, Figure 1A). Correlation analysis indicated positive relationships between FAM66C level and three clinicopathological features, including pathological pattern, infiltration depth and T stage (Table 1). Then, the median score of FAM66C expression was defined as the cutoff value for dividing all patients into high and low expression groups. Kaplan-Meier analysis demonstrated that GC patients with FAM66C high expression exhibited remarkably worse overall survival (OS) and progression-free survival (PFS) compared with those with low expression (All *P* < 0.001, Figures 1B,C). Univariate analysis revealed that TNM stage, pathological pattern, infiltration depth, histological grade, tumor location and FAM66C expression were significantly predictors for OS and PFS of GC patients (All *P* < 0.05, Table 2). Multivariate analysis further demonstrated that FAM66C expression was an independent risk predictor for OS and PFS. In addition, TNM stage and tumor location were also independent predictors for poor prognosis in GC patients (All *P* < 0.05, Table 2). Taken together, these data indicate that FAM66C is upregulated and represents a potential prognostic biomarker in GC patients.

**TABLE 1:** Relationship between the expression level of lncRNA FAM66C and clinicopathological features in GC patients.

Characteristics	Case number N (%)	FAM66C expression		*P* Value
		High (N, %)	Low (N, %)	
Gender	​	​	​	0.776
Female	52 (35.4)	25 (48.1)	27 (51.9)	​
Male	95 (64.6)	48 (50.5)	47 (49.5)	​
Age (years)	​	​	​	0.282
<60	87 (59.2)	40 (46.0)	47 (54.0)	​
≥60	60 (40.8)	33 (55.0)	27 (45.0)	​
Histological grade	​	​	​	0.065
Low differentiation	96 (65.3)	53 (55.2)	43 (44.8)	​
Not-low differentiation	51 (34.7)	20 (39.2)	31 (60.8)	​
Pathological pattern	​	​	​	0.023*
Adenocarcinoma	123 (83.7)	56 (45.5)	67 (54.5)	​
Not-simple adenocarcinoma	24 (16.3)	17 (70.8)	7 (29.2)	​
Infiltration depth	​	​	​	0.005*
Intramucosal/Submucoal/Muscle layer/Subserosa	55 (37.4)	19 (34.5)	36 (65.5)	​
Serosa or adjacent structures	92 (62.6)	54 (58.7)	38 (41.3)	​
Primary site	​	​	​	0.980
Gastric	135 (91.8)	67 (49.6)	68 (50.4)	​
Transgastroesophageal junction/Gastric stump	12 (8.2)	6 (50.0)	6 (50.0)	​
T Stage	​	​	​	0.005*
T1-T3	55 (37.4)	19 (34.5)	36 (65.5)	​
T4	92 (62.6)	54 (58.7)	38 (41.3)	​
N stage	​	​	​	0.105
N0-N1	58 (39.5)	24 (41.4)	34 (58.6)	​
N2-N3	89 (60.5)	49 (55.1)	40 (44.9)	​
M Stage	​	​	​	0.820
M0	130 (88.4)	65 (50.0)	65 (50.0)	​
M1	17 (11.6)	8 (47.1)	9 (52.9)	​
TNM stage	​	​	​	0.154
I	9 (6.1)	5 (55.6)	4 (44.4)	​
II	40 (27.2)	14 (35.0)	26 (65.0)	​
III	81 (55.1)	46 (56.8)	35 (43.2)	​
IV	17 (11.6)	8 (47.1)	9 (52.9)	​

* Represents P values smaller than 0.05; GC, gastric cancer; TNM, tumor node metastasis.

**TABLE 2:** Univariate and multivariate Cox regression analysis of lncRNA FAM66C and survival in GC patients.

Clinical variables	Univariate analysis		P Value	Multivariate analysis		P Value
	HR	95%CI		HR	95%CI	
Overall survival						
Gender (Male vs. Female)	0.757	0.443–1.295	0.310	​	​	​
Age (<60 vs. ≥60)	0.781	0.462–1.321	0.356	​	​	​
Smoking status (Never vs. Ever)	0.849	0.491–1.467	0.558	​	​	​
Drinking status (Never vs. Ever)	0.639	0.343–1.188	0.157	​	​	​
TNM stage (I/II vs. III/IV)	0.282	0.138–0.576	0.001*	0.380	0.180–0.799	0.011*
Pathological pattern (adenocarcinoma vs. Non-simple adenocarcinoma)	0.425	0.232–0.781	0.006*	0.812	0.420–1.571	0.537
Infiltration depth (Intramucosal/Submucosal/Muscle layer/Subserosa vs. Serosa or adjacent structures)	0.315	0.162–0.609	0.001*	0.517	0.256–1.044	0.066
Histological grade (low differentiation vs. Non-simple low differentiation)	1.939	1.058–3.553	0.032*	1.729	0.938–3.186	0.079
FAM66C expression (high vs. low)	2.528	1.468–4.356	0.001*	2.002	1,150–3.483	0.014*
Tumor location (gastric vs. Transgastroesophageal junction/Gastric stump)	0.355	0.173–0.728	0.005*	0.463	0.222–0.967	0.040*
Progression-free survival						
Gender (Male vs. Female)	0.751	0.437–1.288	0.298	​	​	​
Age (<60 vs. ≥60)	0.787	0.465–1.332	0.372	​	​	​
Smoking status (Never vs. Ever)	0.940	0.544–1.625	0.824	​	​	​
Drinking status (Never vs. Ever)	0.691	0.372–1.286	0.244	​	​	​
TNM stage (I/II vs. III/IV)	0.266	0.130–0.545	<0.001*	0.370	0.175–0.785	0.010*
Pathological pattern (adenocarcinoma vs. Non-simple adenocarcinoma)	0.483	0.262–0.891	0.020*	1.375	0.617–3.066	0.436
Infiltration depth (Intramucosal/Submucosal/Muscle layer/Subserosa vs. Serosa or adjacent structures)	0.306	0.158–0.593	<0.001*	0.520	0.256–1.053	0.069
Histological grade (low differentiation vs. Non-simple low differentiation)	1.866	1.016–3.426	0.044*	1.578	0.852–2.922	0.147
FAM66C expression (high vs. low)	2.738	1.587–4.725	<0.001*	2.232	1.284–3.880	0.004*
Tumor location (gastric vs. Transgastroesophageal junction/Gastric stump)	0.362	0.176–0.744	0.006*	0.418	0.197–0.889	0.024*

* Represents P values smaller than 0.05.; TNM, tumour node metastasis; HR, hazard ratio; CI, confidence interval.

### FAM66C overexpression is positively correlative with GC progression and chemotherapy resistance

To explore the clinical significance of FAM66C expression in GC patients, all patients were stratified into different subgroups according to TNM stage and chemosensitivity. When patients were stratified by T stage, survival analysis showed that patients with high FAM66C expression had a significantly shorter OS and PFS rate in T4 stage subgroup (Figure 1E). But in T1-3 stage subgroup, patients with high FAM66C level have only poorer PFS (not OS) than those with low level (Figure 1D). Furthermore, we also found that high expression of FAM66C was associated with worse OS and PFS in patients with N2-3 stage (Figure 1G), M0 stage (Figure 1H), but not in those with N0-1 stage (Figure 1F) and M1 stage (Figure 1I).

Given that FAM66C was initially identified in chemoresistant GC cells by bioinformatics analysis (Yang et al., 2022), we further observed the relationship between this lncRNA and GC chemosensitivity. We selected 100 patients who had relatively detailed documentation about their chemotherapy from our cohort. Subsequently, these patients were divided into two subgroups, patients resistant to chemotherapy and patients sensitive to chemotherapy. Interestingly, in the subgroup with patients resistant to chemotherapy, patients with high FAM66C expression had significantly poorer OS and PFS than those with low expression (Figure 1J). But there was no significant statistical difference in patients sensitive to chemotherapy (Figure 1K). Collectively, these findings underscore an important role of FAM66C in GC progression and chemoresistance.

### Increased expression of FAM66C elicits the proliferation and chemoresistance of GC cells

**FIGURE 2:**
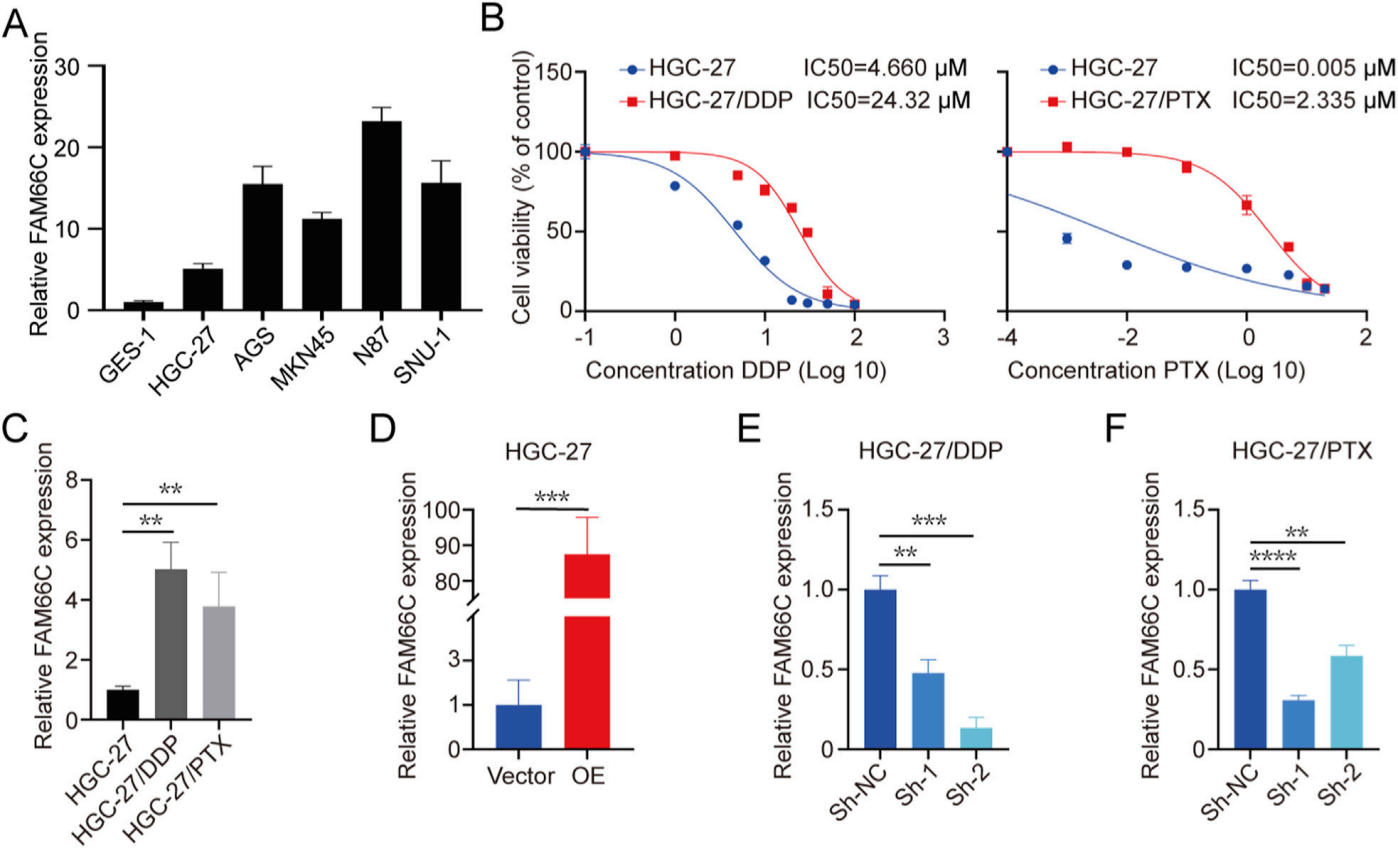
Construction of cisplatin-resistant gastric cancer cell lines. **(A)** RT-PCR analyses of FAM66C expression in 5 GC cell lines and normal gastric epithelia cell line GES-1. **(B)** IC50 assay of DDP-resistant GC cell lines and their parental cells. **(C)** RT-PCR analyses of FAM66C expression in HGC-27/DDP, HGC-27/PTX and their parental cell line HGC-27 cells. **(D)** RT-PCR analyses was performed to observe the expression of FAM66C in HGC-27 and HGC-27 with FAM66C overexpression. **(E,F)** Relative expression of FAM66C was confirmed by RT-PCR in HGC-27/DDP and HGC-27/PTX cells transfected with two independent shRNA targeting FAM66C. Data are presented as mean ± SEM, n = 3. **P < 0.01, ***P < 0.001, ****P < 0.0001.

We also performed RT-PCR to determine the expression of FAM66C in GC cell lines. The result indicated FAM66C expression was commonly higher in 5 GC cell lines than in the normal gastric epithelia cell line GES-1 (Figure 2A). Moreover, after continuous exposure to increasing concentration of cisplatin (DDP) or paclitaxel (PTX) for more than 1 year, we successfully generated DDP-resistant GC cell line HGC-27/DDP and PTX-resistant GC cell line HGC-27/PTX. The IC50 value for HGC-27/DDP and HGC-27/PTX cells were 24.32 μmol/L and 2.335 μmol/L, which were higher than their parent cells, respectively (Figure 2B). RT-PCR assay confirmed FAM66C expression was upregulated in these two chemoresistant GC cells compared to their parent cells (Figure 2C). Therefore, we selected HGC-27 cells with lower FAM66C expression for FAM66C overexpression, and HGC-27/DDP and HGC-27/PTX cells with higher FAM66C expression for FAM66C knockdown. Successfully knockdown or overexpression of FAM66C was verified by RT-PCR (Figures 2D–F).

**FIGURE 3:**
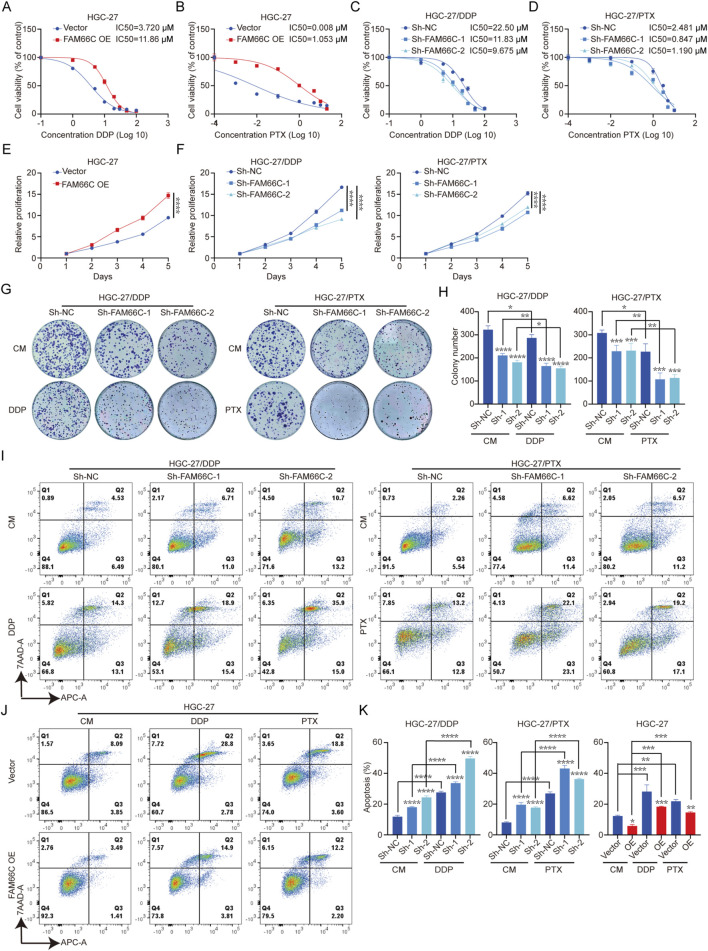
Increased expression of FAM66C promoted the proliferation and chemoresistance of GC cells. **(A,B)** Measurement of IC50 cytotoxicity assay for DDP or PTX in HGC-27 cells with FAM66C overexpression. **(C,D)** Measurement of IC50 cytotoxicity assay for DDP or PTX in HGC-27/DDP and HGC/PTX cells after knockdown of FAM66C, respectively. **(E,F)** CCK8 assay of HGC27 cells with FAM66C overexpression and HGC27/DDP or HGC27/PTX cells with FAM66C inhibition. **(G,H)** Representative results of the colony formation assay of HGC27/DDP or HGC27/PTX cells after Sh-FAM66C-1 and Sh-FAM66C-2 transfection treated with conditional medium (CM) and DDP or PTX. Representative images of flow cytometric analyses of DDP/PTX-induced apoptosis in HGC27/DDP or HGC27/PTX cells after knockdown of FAM66C **(I)**, and in HGC27 cells after upregulation of FAM66C **(J)**. **(K)** Quantifications of cell apoptosis (consists of early and late apoptotic cells) were shown. Q1 represents dead cells, Q2 represents late apoptotic cells, Q3 represents early apoptotic cells and Q4 represents alive cells. Error bars: mean ± SD from three independent experiments. *P < 0.05, **P < 0.01, ***P < 0.001, ****P < 0.0001.

Subsequently, gain- and loss-of-function experiments were conducted to investigate the influence of FAM66C on proliferation and chemoresistance. Elevated expression of FAM66C increased the IC50 value of HGC-27 cells to DDP and PTX (Figures 3A,B). Conversely, knockdown of FAM66C sensitized HGC-27/DDP and HGC-27/PTX cells to chemotherapy, as evidenced by a 50%–66.7% reduction in IC50 values (Figures 3C,D). Furthermore, CCK8 and colony formation assays showed that upregulation or downregulation of FAM66C enhanced or inhibited GC proliferation (Figures 3E–H). After DDP or PTX treatment, the colony numbers were more markedly decreased in HGC-27/DDP and HGC-27/PTX cells with FAM66C silencing (Figures 3G,H). Flow cytometric assays obtained similar results, whereas FAM66C overexpression resulted in the opposite effects in HGC-27 cells (Figures 3I–K). Additionally, we investigated colony formation or apoptosis of these GC cells with FAM66C knockdown between CM and DDP and CM and PTX. The results showed that compared with conditional medium (CM), knockdown of FAM66C could sensitize GC cells to theses chemotherapy drugs (Figures 3H,K). Therefore, these findings suggest that FAM66C promotes GC growth and induces chemoresistance *in vitro*.

### FAM66C promotes GC growth and chemoresistance *in vivo*

**FIGURE 4:**
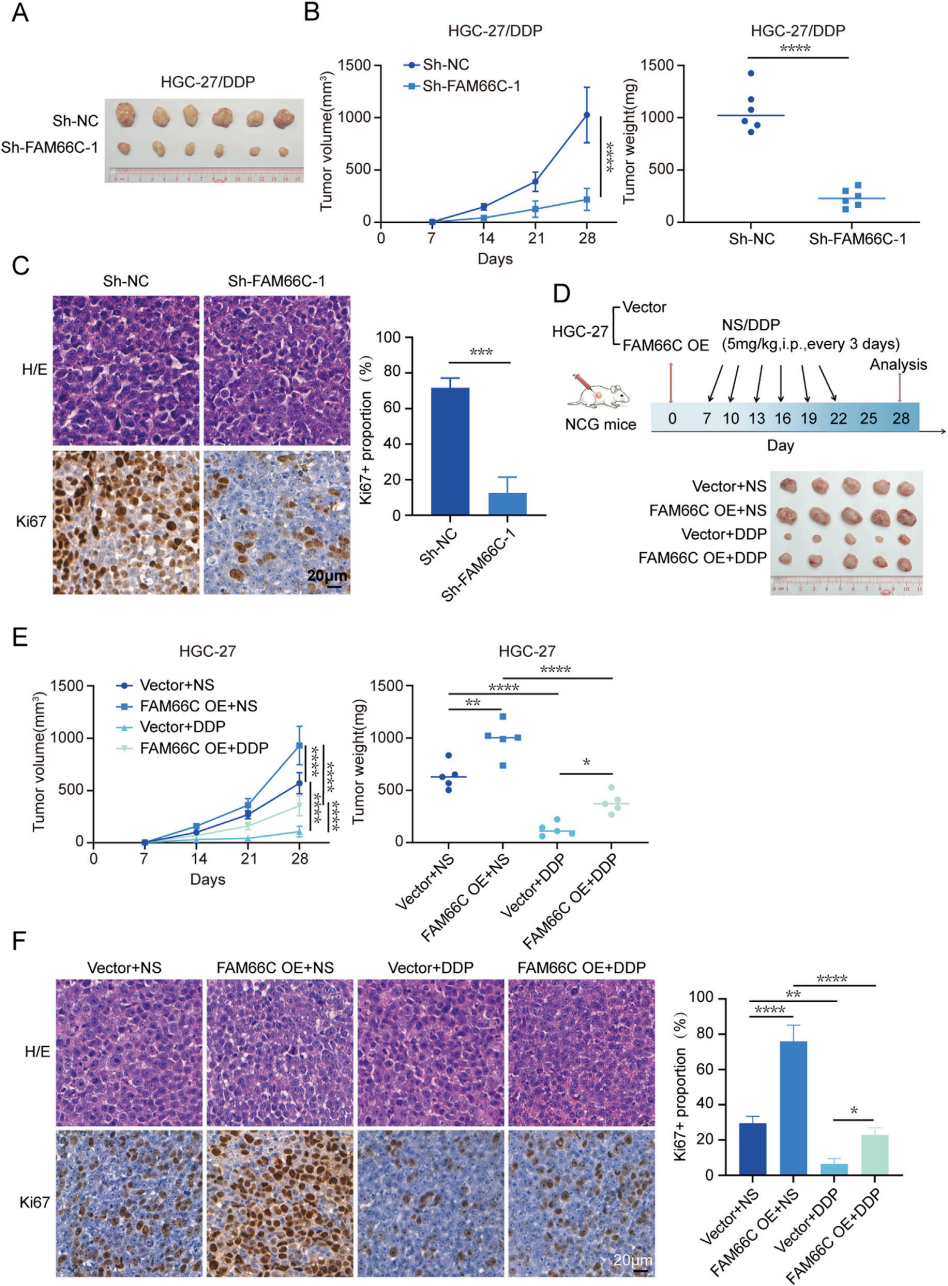
FAM66C enhances GC growth and chemoresistance in xenograft mouse models. **(A)** HGC27/DDP cells transfected with an empty vector and cells transfected with a shRNA targeting FAM66C were inoculated into NGC mice. Each group has six mice. After 4 weeks of injection, the size of tumors isolated from both groups were measured using a straightedge. **(B)** Tumor volume and weight of the xenograft in FAM66C inhibition group and NC group. **(C)** Representative images and quantification of H&E and immunohistochemical analyses of the expression of Ki67 in two groups. HGC27 cells stably expressing FAM66C and vector were inoculated into NGC mice. Each group has five mice. After 6 days of injection, normal saline (NS) or DDP (5 μg/mg) was administered by intraperitoneal injection every 3 days. Representative tumor images **(D, upper panel)**, tumor size **(D, bottom panel)**, tumor volume and weight **(E)** were shown. **(F)** Representative images and quantification of H&E and immunohistochemical analyses of Ki67 expression in different groups. Original magnification: ×400. Scale bar = 20 μm. Data represent the mean ± SD. *P < 0.05, **P < 0.01, ***P < 0.001, ****P < 0.0001.

To observe the proliferative and chemoresistant potential of FAM66C *in vivo*, we established xenograft mouse models by subcutaneously injecting HGC-27/DDP cells with stable FAM66C knockdown. The results showed that FAM66C knockdown significantly inhibited tumor growth compared to control group (Figure 4A), and the tumor volume and weight were decreased distinctly (Figure 4B). Immunohistochemical (IHC) analysis described that Ki67 expression in FAM66C knockdown group was strikingly reduced than control group (Figure 4C). Take a step further, we observed the effect of FAM66C overexpression on the chemosensitivity of GC *in vivo*. As shown in Figure 4D, we subcutaneously injected HGC-27 cells with stable FAM66C overexpression into nude mice followed by DDP treatment. We found that ectopic FAM66C expression potentiated tumorigenic capacity compared to control group (Figure 4D). Significantly decreased tumor volume and weight were observed in normal mice treated with DDP, but the effect was reversed by FAM66C upregulation (Figures 4D,E). IHC analysis indicated that FAM66C stimulated Ki67 expression in xenograft tumors, which was effectively counteracted by DDP treatment (Figure 4F). Thus, the aforementioned data of the *in vitro* and *in vivo* experiments strongly suggest that FAM66C is a novel oncogene in GC progression and chemoresistance.

### FAM66C promotes YAP phosphorylation in the nucleus through a direct interaction, independently of the hippo pathway

**FIGURE 5:**
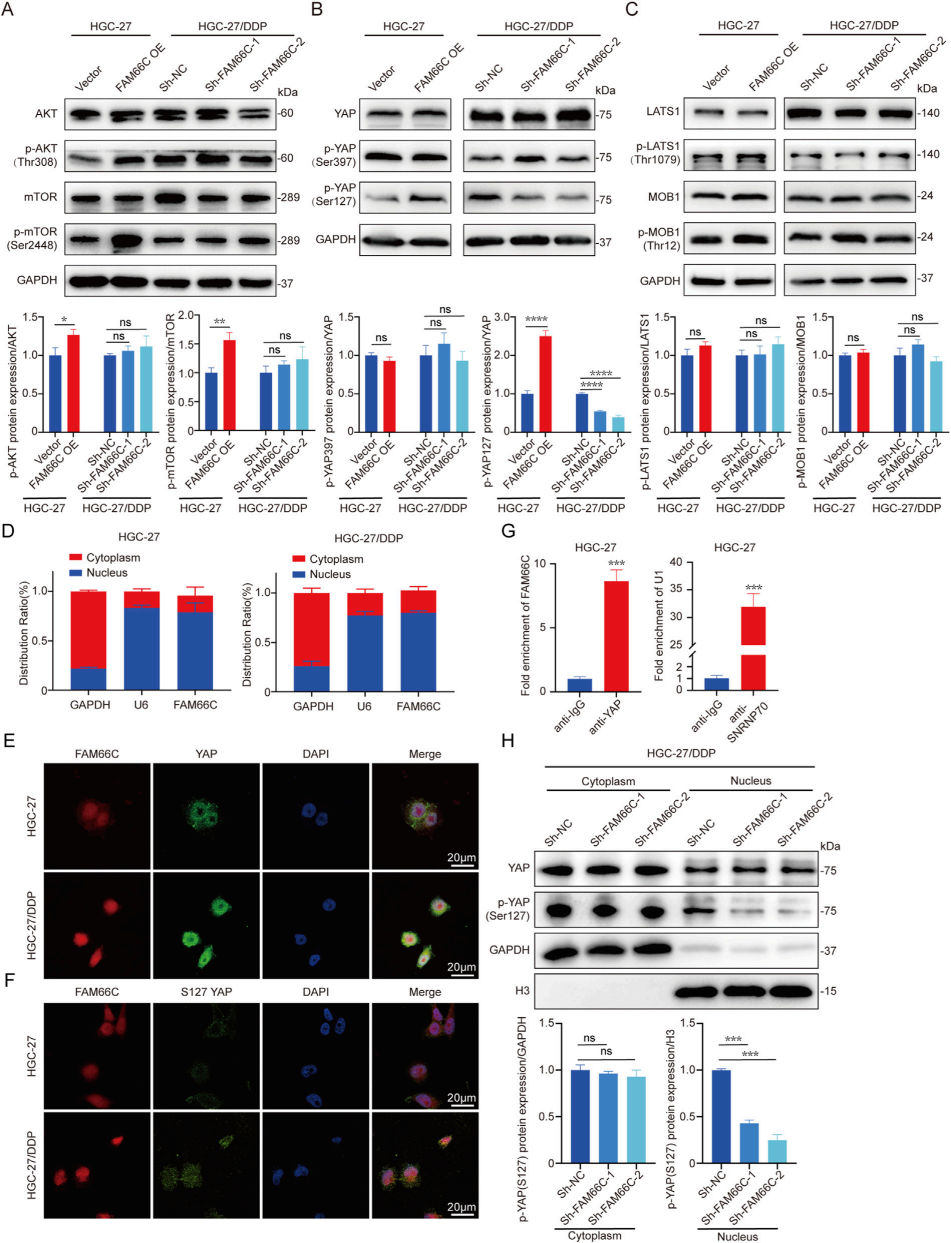
FAM66C promotes nuclear YAP phosphorylation via interacting with nuclear YAP. **(A)** Western blotting of investigating the expression of key proteins of PI3K/AKT/mTOR and Hippo pathways in HGC-27 cells with FAM66C overexpression and HGC-27/DDP cells after knockdown of FAM66C. **(B)** Phosphorylated YAP protein (S127, S297) expression in abovementioned GC cells were detected by Western blotting. **(C)** Immunoblotting analysis of the expression of the upstream proteins (p-LATS1 and p-MOB1) of Hippo signaling pathway in abovementioned GC cells. **(A–C)** For the quantitative analysis of protein levels, band density was evaluated using ImageJ software and represented as relative intensity. **(D)** Nuclear-cytoplasmic fractionation assay of the subcellular localization of FAM66C in HGC-27 and HGC27/DDP cells. **(E, F)** Representative images of IF micrographs of the subcellular localization and expression of FAM66C (red) and YAP or S127 YAP (green) in HGC-27 and HGC27/DDP cells. Nuclei were counterstained with DAPI (blue). Scale bars represent 20 μm. **(G)** RIP assay for the interaction between YAP protein and FAM66C in HGC-27 cell. SNRNP70 and U1 were positive control. **(H)** Cytoplasmic and nuclear expression of phosphorylated YAP protein (S127, S297) expression in HGC27/DDP cells with FAM66C inhibition. For the quantitative analysis of p-YAP(S127) protein level, band density was evaluated using ImageJ software and represented as relative intensity. GAPDH was used as the control for the cytoplasmic protein. Histone H3 was used as the control for the nuclear protein. Data are represented as the mean ± SD, n = 3. ns, not significant; **P < 0.01, ***P < 0.001.

It is increasingly recognized that lncRNAs could regulate tumor chemoresistance through scaffolding multiple transcription related proteins or interacting with transcription factors (Eptaminitaki et al., 2022). Therefore, we firstly investigated the expression of key proteins of PI3K/AKT/mTOR and Hippo pathways. We found that upregulation of FAM66C significantly increased p-AKT and p-mTOR expression, but there were no significant differences in p-AKT and p-mTOR expression after FAM66C knockdown (Figure 5A). Interestingly, loss of FAM66C contributed to decrease the expression of YAP phosphorylation at S127 (not S397), and enhancing expression of FAM66C led to an opposite result (Figure 5B). Whereas, no significant changes in the expression of upstream proteins (p-LATS1 and p-MOB1) of Hippo signaling pathway were observed following aberrant expression of FAM66C (Figure 5C). These findings suggested that FAM66C regulates YAP phosphorylation independently of the canonical Hippo pathway.

Previous study demonstrated that cyclin-dependent kinase 7 (CDK7) regulated YAP expression by phosphorylating nuclear YAP independently of Hippo pathway (Lv et al., 2023). Therefore, we hypothesized that FAM66C might regulate YAP expression in a manner similar to CDK7. To verify the hypothesis, the nuclear-cytoplasmic fractionation assay confirmed the predominant nuclear distribution of FAM66C in HGC-27 and HGC-27/DDP cells (Figure 5D). RNA FISH assay indicated that FAM66C co-localized with YAP and S127 YAP in the nucleus of GC cells (Figures 5E,F). Furthermore, the interaction between FAM66C and YAP in GC cells was determined by RIP analysis. The result demonstrated that FAM66C physically interacted with nuclear YAP protein (Figure 5G). FAM66C knockdown significantly inhibited YAP phosphorylation (S127) in the nucleus but not the cytoplasm of GC cells (Figure 5H). Collectively, these data revealed that FAM66C regulates nuclear YAP phosphorylation via interacting with nuclear YAP in a Hippo pathway-independent manner.

### YAP depletion contracts FAM66C-induced proliferation and chemoresistance in GC cells

**FIGURE 6:**
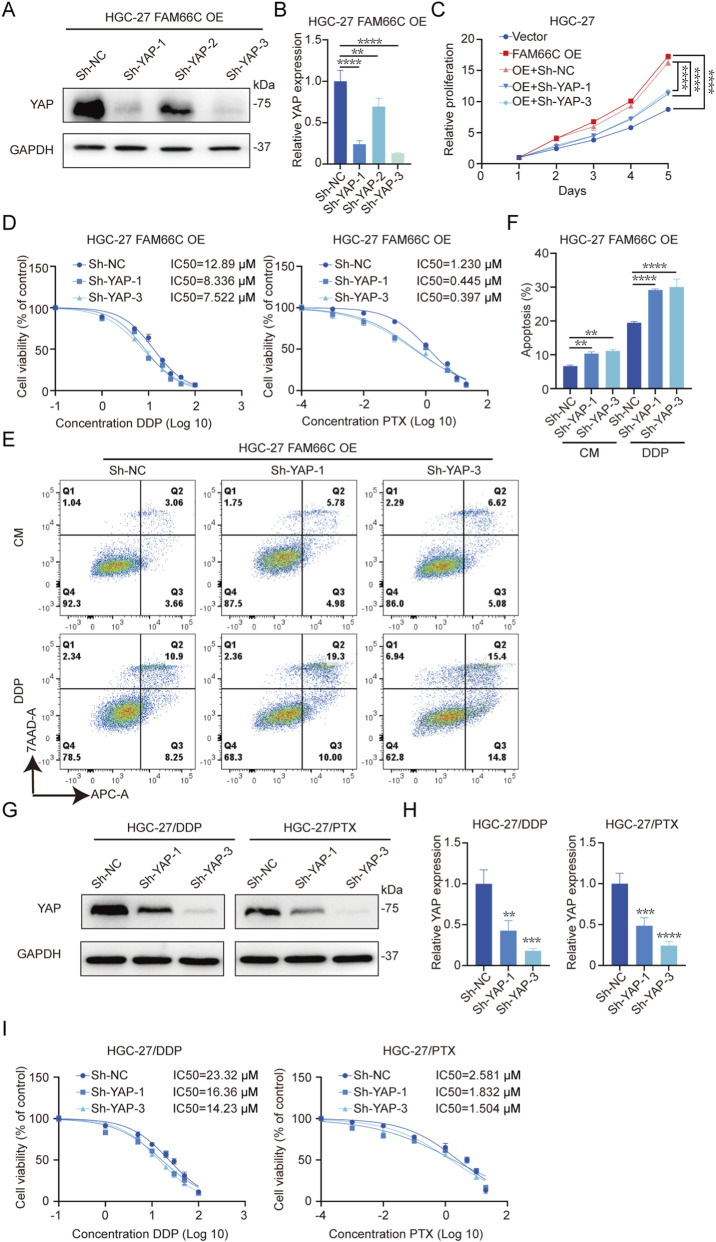
Reduced expression of YAP rescues the activator function of FAM66C in GC proliferation and chemoresistance. Relative expression of YAP confirmed by Western blotting **(A)** and RT-PCR **(B)** in HGC-27 cells with FAM66C overexpression transfected with three independent shRNA targeting YAP. **(C)** CCK8 assay of HGC27 cells with FAM66C overexpression after Sh-YAP-1 or Sh-YAP-3 transfection. **(D)** IC50 cytotoxicity assay for DDP or PTX in HGC-27 cells with FAM66C overexpression transfected with Sh-YAP-1 or Sh-YAP-3. **(E,F)** Representative images and quantification of flow cytometric analyses of DDP/PTX-induced apoptosis in abovementioned GC cells. **(G,H)** Western blotting and RT-PCT of investigating YAP expression in HGC27/DDP or HGC27/PTX cells with YAP knockdown. **(I)** IC50 assay for DDP or PTX in HGC27/DDP or HGC27/PTX cells transfected with Sh-YAP-1 or Sh-YAP-3. For all quantitative results, the data are represented as the mean ± SD from three independent experiments. **P < 0.01, ***P < 0.001, ****P < 0.0001.

To determine whether YAP protein is involved in FAM66C regulation of GC chemoresistance, YAP was artificially downregulated in HGC-27 cells with FAM66C overexpression, and the efficient knockdown of YAP was verified by Western blotting and RT-PCR (Figures 6A,B). CCK8 and IC50 assays were subsequently conducted. The results indicated that YAP knockdown significantly inhibited tumor proliferation and reduced the resistance of GC cells to DDP and PTX (Figures 6C,D). Apoptosis assay showed that ablation of YAP effectively reversed the protective effect of FAM66C on GC cells upon chemotherapy, showing approximately 1.5-fold increase in DDP-induced apoptosis (Figures 6E,F). We also used shRNA to silence YAP expression in HGC-27/DDP and HGC-27/PTX cells, and effective knockdown of YAP in both cell lines were confirmed by Western blotting and RT-PCR (Figures 6G,H). IC50 assay showed that inhibition of YAP sensitized chemoresistant GC cells to DDP or PTX (Figure 6I). Taken together, these rescue experiments suggest that under-expression of YAP reversed FAM66C-induced proliferation and chemoresistance in GC cells.

## Discussion

Extensive published evidence has been demonstrated that a series of lncRNAs have major roles in GC diagnosis, prognosis and chemoresistance (Yuan et al., 2020). FAM66C has been identified as a potential biomarker and regulator in the progressioin of several cancers (Xie et al., 2020; Lei et al., 2021; Wang et al., 2023). However, the expression and biological function of FAM66C in GC are poorly understood. Our previous study showed that this lncRNA was implicated in GC chemotherapy resistance (Yang et al., 2022). But it remains unclear whether FAM66C contributes to the chemoresistance of GC cells. In this study, we observed FAM66C overexpression in GC tissues, and its expression was significantly correlated with tumor progression and chemoresistance in GC patients. Moreover, FAM66C exerted a pro-proliferative and pro-chemoresistant effects in GC cells, as demonstrated by both *in vitro* and *in vivo* experiments. Consequently, FAM66C represents a potential diagnostic and prognostic biomarker and a promising therapeutic target for overcoming GC chemoresistance.

Growing evidence has pointed out that lncRNAs regulated GC chemoresistance via several signaling pathways, including AKT/mTOR, Wnt/β-catenin and Hippo pathways (Yuan et al., 2020). Unfortunately, we found that p-AKT and p-mTOR expression were significantly increased in HGC-27 cells after FAM66C overexpression, but there were no significant statistical differences in p-AKT and p-mTOR expression between HGC27/DDP cells expressing Sh-NC and Sh-FAM66C. A possible explanation for these contradictory results of FAM66C gene may be due to the functional redundancy or compensatory mechanisms of PI3K/AKT/mTOR pathway. Additionally, another possible explanation is that FAM66C may act indirectly to regulate AKT or mTOR phosphorylation by regulating other genes. Therefore, FAM66C may promote GC chemoresistance by regulating an alternative signaling pathway.

It is well-established that the hyperactivation of Hippo pathway contributes to the aggressive phenotype of GC cells (Ajani et al., 2021; Chen et al., 2023; Messina et al., 2023). Recent studies have shown the involvement of lncRNAs in Hippo/YAP-induced tumor progression. For example, PR11-323N12.5 promoted GC proliferation and immunosuppression through increasing YAP1 transcription (Wang et al., 2021). ELFN1-AS1 had a tumor-promoting effect by interacting with TAOK1 to inhibit Hippo pathway (Wang et al., 2024). MIR181A2HG enhanced GC lymph node metastasis by upregulating versican (VCAN) to activate Hippo pathway (Zang et al., 2025). These studies suggest that Hippo signaling pathway can serve as the downstream effector of lncRNAs in GC progression. Herein, we demonstrated for the first time that FAM66C promotes GC chemoresistance by phosphorylating YAP protein in a Hippo pathway-independent manner. Similarly, Chen et al. (Chen et al., 2017) reported that lincRNA-p21 elevated YAP expression and promoted its nucleus translocation independently of the Hippo pathway. However, this study did not elucidate how lincRNA-p21 regulates YAP expression in GC. Interestingly, CDK7 interacted with nuclear YAP and phosphorylated YAP at its S127 and S397 in a Hippo pathway-independent manner (Lv et al., 2023). In addition, ULK1/2 translocated into nucleus and stabilized nuclear YAP by phosphorylating its S227 in response to hypoxia (Jia et al., 2021). Therefore, we hypothesized that YAP phosphorylation mediated by FAM66C in GC did not rely on Hippo pathway. Our subsequent experiments confirmed that FAM66C interacted with nuclear YAP and phosphorylated it at S127, resulting in GC proliferation and chemoresistance. Collectively, these data indicate that FAM66C facilitated nuclear YAP expression and phosphorylation in a Hippo pathway-independent manner.

In recent years, a growing body of evidence has provided support for noncanonical YAP/TAZ activation mechanism independent of the Hippo pathway (Zhang et al., 2025). Our findings first indicated that lncRNA FAM66C modulated nuclear YAP in a Hippo-independent manner in GC chemoresistance. However, the mechanism by which lncRNAs mediate non-canonical YAP/TAZ activation in cancers remains poorly understood. Future study will explore the underlying mechanism of FAM66C upregulation in GC chemoresistance. We speculate that FAM66C overexpression may result from genomic amplification, epigenetic alterations such as DNA methylation, or dysregulation of specific transcriptional factors. Additionally, recent studies indicated that nuclear YAP phosphorylation facilitated PKM2 and LDHD transcription, and then promoted tumor proliferation (Jia et al., 2021; Lv et al., 2023). Thus, we will investigate the downstream target genes of phosphorylated YAP, such as PKM2 and LDHD, in chemoresistant GC cells.

Our study has several limitations. First, the clinical data analysis may be subject to bias due to the limited number of GC tissue samples. Thus, we will expand cohort size (more than 300) to validate the clinical results of this study. Second, we also established another chemoresistant GC cell line from MGC-803 parent cells. In the future, we will observe the impact of aberrant expression of FAM66C on this model. Third, the precise mechanism by which FAM66C confers GC chemoresistance requires further elucidation. Future study will explore the underlying interaction between FAM66C and YAP protein, and evaluate potential downstream effectors of YAP in GC cells.

In summary, we reported that FAM66C was overexpressed in GC tissues and it had proliferation- and chemoresistance-promoting effects in GC cells. We further revealed a novel mechanism in which FAM66C interacted with and phosphorylated nuclear YAP in a Hippo-independent manner, thereby promoting GC chemoresistance. Therefore, FAM66C might be a promising prognostic biomarker for GC patients, and provide a basis for developing FAM66C-targeted therapies which could overcome GC chemoresistance.

## Data Availability

The original contributions presented in the study are included in the article/supplementary material, further inquiries can be directed to the corresponding author.
